# Pharmacological Activation of GPR68 Attenuates Ferroptosis in Spinal Cord Ischemia/Reperfusion Injury Through PI3K/Akt-Mediated Nrf2 Antioxidant Pathway

**DOI:** 10.1007/s10753-025-02326-0

**Published:** 2025-06-18

**Authors:** Ruitong Yang, Jintao Ye, Pengbo Wang, Tao Liu, Bin Cheng, Fengtao Li

**Affiliations:** 1 Second Affiliated Hospital of Xi’an Jiaotong University, No.157 Xiwu Road, Xi’an, 710000 Shaanxi China; 2 Xi’an Jiaotong University, No. 74 Yanta West Road, Xi’an, 710000 Shaanxi China

**Keywords:** GPR68, Ferroptosis, Spinal cord injury, Ischemia-reperfusion, PI3K/Akt-Nrf2 axis

## Abstract

Spinal cord ischemia-reperfusion injury (SCIRI) is a devastating condition with limited therapeutic options. This study unveils a novel role of G protein-coupled receptor 68 (GPR68), a pH-sensing G protein-coupled receptor (GPCR), in mitigating ferroptosis—a lipid peroxidation-driven cell death—through the Phosphoinositide 3-Kinase/ Protein Kinase B/ Nuclear Factor Erythroid 2-Related Factor 2 (PI3K/Akt/Nrf2) antioxidant axis. Using in vitro (Oxygen-Glucose Deprivation/Reperfusion (OGD/R)-treated Pheochromocytoma Cell Line 12 (PC12) cells ) and in vivo (rat spinal cord ischemia-reperfusion (I/R) ) models, we demonstrate that GPR68 downregulation exacerbates ferroptosis, evidenced by elevated Acyl-CoA Synthetase Long-Chain Family Member 4 (ACSL4), Malondialdehyde (MDA), and Oxidized Glutathione/ Total Glutathione (GSSG/T-GSH) levels, alongside reduced Solute Carrier Family 7 Member 11 (SLC7A11) and Glutathione Peroxidase 4 (GPX4). Pharmacological activation of GPR68 with MS48107 or the clinically approved benzodiazepine Lorazepam robustly reversed ferroptosis by enhancing Akt phosphorylation and Nrf2 nuclear translocation. Mechanistically, GPR68 siRNA or PI3K/Akt inhibition abolished these protective effects. Crucially, Lorazepam rescued neuronal viability and suppressed ferroptosis in spinal I/R rats, effects fully negated by the GPR68 antagonist Ogremorphin (OGM). Our findings establish GPR68 as a key ferroptosis regulator and propose repurposing Lorazepam as a therapeutic strategy for SCIRI.

**Supplementary Information:**

The online version contains supplementary material available at 10.1007/s10753-025-02326-0.

## Introduction

SCIRI is a critical complication following spinal surgeries or traumatic injuries, often causing irreversible neurological deficits [1–3]. The pathophysiology involves energy failure during ischemia and oxidative stress during reperfusion, with local acidosis exacerbating cellular damage through anaerobic glycolysis [4–6]. Despite advances in understanding ferroptosis—an iron-dependent cell death driven by lipid peroxidation—the upstream regulators linking microenvironmental acidosis to ferroptosis cascades remain elusive [7–9].

GPR68, a proton-sensing GPCR enriched in neural tissues, emerges as a key candidate. While GPR68 is known to modulate calcium flux and inflammatory signaling in acidosis [10–12], its role in ferroptosis regulation is unexplored. Studies have demonstrated that among pancreatic cancer patients undergoing chemotherapy, the use of lorazepam is associated with worsened progression-free survival (PFS), whereas alprazolam exhibits no such correlation. Researchers hypothesize this discrepancy may be linked to lorazepam’s agonistic activity on GPR68. The study further revealed elevated GPR68 expression in pancreatic ductal adenocarcinoma (PDAC), potentially elucidating the mechanism underlying lorazepam-mediated tumor-protective effects [13]. Similarly, separate investigations indicate that pharmacological inhibition of GPR68 with OGM induces glioblastoma ferroptosis [14, 15]. Furthermore, studies have demonstrated that GPR68 deficiency enhances radiotherapy sensitivity and promotes ferroptosis across multiple tumor types [16]. These findings collectively suggest a critical role of GPR68 in modulating ferroptosis. Intriguingly, PI3K/Akt activation by GPR68 has been reported in ischemic brain models [17, 18], and Akt-dependent Nrf2 nuclear translocation is a canonical antioxidant mechanism [19–22]. The relationship between the Akt-Nrf2 pathway and SCIRI involves critical regulatory mechanisms that influence oxidative stress, inflammation, apoptosis, and neuronal survival following ischemic insult and subsequent reperfusion. Akt activation plays a pivotal role in mitigating ischemia-reperfusion injury by suppressing apoptotic pathways, enhancing cell survival signals, and promoting Nrf2 nuclear translocation [23]. Nrf2, a master regulator of antioxidant defense [24], binds to antioxidant response elements (ARE) to upregulate genes encoding detoxifying enzymes (e.g., HO-1, NQO1) and reactive oxygen species (ROS)-scavenging proteins, thereby counteracting oxidative damage induced by reperfusion [25]. The PI3K/Akt pathway positively modulates Nrf2 activity, creating a synergistic protective effect that reduces neuronal death, attenuates inflammatory cytokine release (e.g., TNF-α, IL-1β, IL-6), and inhibits excessive ROS production during SCIRI [26, 27]. Studies suggest that pharmacological activation of Akt-Nrf2 signaling—through natural compounds like polydatin or ginsenosides—can ameliorate SCIRI by enhancing antioxidant capacity, suppressing NF-κB-mediated neuroinflammation, and preserving mitochondrial function. However, the therapeutic potential of these agents is limited by poor bioavailability, necessitating advanced drug delivery systems to optimize CNS penetration and sustained efficacy [28]. Thus, targeting the Akt-Nrf2 axis represents a promising strategy to mitigate ischemia-reperfusion damage, balancing neuroprotection and functional recovery in SCIRI. Furthermore, whether GPR68 orchestrates the PI3K/Akt/Nrf2 axis to suppress ferroptosis in SCIRI is unknown—a critical gap given the therapeutic potential of pH-sensitive receptors.

Here, we hypothesize that GPR68 downregulation exacerbates SCIRI by disabling Akt/Nrf2-mediated ferroptosis suppression, and that pharmacological GPR68 activation rescues this pathway. Through systematic investigation in PC12 cells [29, 30] and a rat spinal cord I/R model, we establish that GPR68 activation protects against ferroptosis via hierarchical PI3K/Akt/Nrf2 signaling. In vitro, OGD/R-induced ferroptosis coincided with GPR68 downregulation, while GPR68 Positive Allosteric Modulator compound (MS48107) [31] reversed lipid peroxidation through Akt phosphorylation and subsequent Nrf2 nuclear translocation—effects fully abolished by GPR68 siRNA or PI3K/Akt inhibition. In vivo, spinal cord I/R injury triggered time-dependent GPR68 depletion concurrent with ferroptosis progression, mirrored by histological degeneration. Crucially, the benzodiazepine Lorazepam—identified as a GPR68-targeted agent [32, 33]—rescued neuronal viability through GPR68-dependent PI3K/Akt/Nrf2 reactivation, with its therapeutic effects completely negated by co-administration of the GPR68 antagonist OGM [31]. These findings collectively define a novel GPR68-PI3K/Akt-Nrf2 axis as a central regulatory node in ferroptosis pathophysiology and provide preclinical validation for repurposing Lorazepam to mitigate SCIRI.

## Materials and Methods

### Animal Models and Modeling Procedures

Male Sprague-Dawley (SD) rats (8 weeks old, 250–300 g) were purchased from the Experimental Animal Center of Xi’an Jiaotong University and used in this study.

SCIRI model was established by transiently clamping the abdominal aorta. Rats were anesthetized with 2% isoflurane (MedChemExpress, Cat No.HY-A0134) in oxygen (2 L/min) and placed in a supine position. After a midline laparotomy, the abdominal aorta was exposed and clamped just below the left renal artery using a non-traumatic vascular clip for 30 min. The clip was then removed to allow reperfusion for 12 h, 1 d, or 3 d, depending on the experimental design: SD rats were randomly assigned to seven groups (*n* = 5 per group): (1) Sham group (surgery without aortic clamping); (2) I/R 12 h group (30 min ischemia + 12 h reperfusion); (3) I/R 1 d group (30 min ischemia + 24 h reperfusion); (4) I/R 3 d group (30 min ischemia + 72 h reperfusion); (5) I/R 3 d + OGM group; (6) I/R 3 d + Lorazepam group; and (7) I/R 3 d + Lorazepam + OGM group. Drug administration protocols (dose, timing, and route) are detailed in the Pharmacological Interventions section.

Postoperative care included monitoring of body temperature (maintained at 37 ± 0.5 °C using a heating pad), observation of motor function recovery, and manual bladder compression every 12 h to assist urination. Rats with severe complications, such as death, were excluded from further analysis. Lumbar spinal cord tissues were collected immediately after the experimental procedures and processed accordingly for different analytical purposes.

### Cell Culture and Oxygen-Glucose Deprivation/Reperfusion (OGD/R) Model

The rat adrenal pheochromocytoma PC12 cell line was purchased from the Shanghai Cell Bank of the Chinese Academy of Sciences (Catalog Number: TCR8) and has passed quality control. The experimental use was restricted to passages 3 to 10. The PC12 cell line was cultured in high-glucose DMEM/F12 medium supplemented with 10% fetal bovine serum and 1% penicillin-streptomycin. Cells were maintained at 37 °C in a humidified incubator with 5% CO₂.

For the OGD/R model, cells were washed with PBS buffer and then incubated in glucose-free, serum-free DMEM/F12 medium for 3 h. During the final 1 h of this period, cells were transferred to a tri-gas incubator with 94% N₂, 5% CO₂, and a controlled O₂ concentration below 1% to induce hypoxia. After the OGD treatment, cells were reperfused for 6 h in low-glucose medium (1.5 g/L glucose) under normoxic conditions (5% CO₂, 95% air).

### SiRNA Knockdown of GPR68 in PC12 Cells

To knock down GPR68 expression in PC12 cells, siRNA targeting GPR68 (sequences: sense 5’-GCAGAUCAAGGCCGAAAUTT-3’, antisense 5’-AUUUGGGCCUUGAUCUGCTT-3’; purchased from Sangon Biotech) was transfected into cells using Lipofectamine 2000 (Thermo Fisher Scientific) following the manufacturer’s protocol. Briefly, PC12 cells were seeded in 6-well plates and transfected with the siRNA at an optimized concentration. After incubation for 48 h, the knockdown efficiency was confirmed by qPCR and Western blotting.

### RNA Extraction and qPCR Analysis

Total RNA was extracted from tissues and cells using Trizol reagent. RNA concentration and purity were assessed using a NanoDrop spectrophotometer, and RNA integrity was confirmed by agarose gel electrophoresis. cDNA was synthesized from 1 µg of total RNA using a reverse transcription kit (Servicebio) according to the manufacturer’s instructions. qPCR was performed using a 2× Universal Blue SYBR Green qPCR Master Mix (Servicebio) on a real-time PCR system (Applied Biosystems). The thermal cycling conditions were carried out according to the manufacturer’s protocol. Gene expression levels were normalized to the housekeeping gene β-actin using the 2^(-ΔΔCt) method.

The primers used for qPCR are listed as follows:

- ACSL4:
- Forward: 5’- ATTGCTGCCTGTCCACTTGT-3’;
- Reverse: 5’- ACAGCTTCTTTGCCAAGGGT-3’;
- SLC7A11:
- Forward: 5’- TCTGGAGGTCTTTGGTCCCT-3’;
- Reverse: 5’- CCTCGGCGCTAATGGTTGTA-3’;
- GPX4:
- Forward: 5’- AACAGCCACGAGTTCCTGG-3’;
- Reverse: 5’- GAGATAGCACGGCAGGTCC-3’;
- GPR68:
- Forward: 5’- CCGAAATGAGCTGGGAGTGT-3’;
- Reverse: 5’- AAGCCAGGAAGATGACCACG-3’;
- ACTIN (β-actin):
- Forward: 5’- TACAGCTTCACCACCACAGC-3’;
- Reverse: 5’- CGGACTCATCGTACTCCTGC-3’;
- NRF2:
- Forward: 5’- TCAGTCTTCACCACCCCTGA-3’;
- Reverse: 5’- TGGGACTTGTGTTCAGCGAA − 3’.

### Western Blot Analysis

Proteins were extracted from tissues and cells using a Nuclear and Cytoplasmic Protein Extraction Kit (Beyotime). Protein concentrations were normalized using a BCA Protein Assay Kit (Servicebio). Western blot analysis was performed using SDS-PAGE and 0.45 μm PVDF membranes (Millipore). Membranes were blocked with Rapid Blocking Buffer (Servicebio) for 10 min at room temperature. The primary antibodies used were ACSL4 (1:600, Solarbio, Cat No. K109571P), SLC7A11 (1:800, Proteintech Group, Cat No.32384-1-AP), GPX4 (1:800, Proteintech Group, Cat No.30388-1-AP), GPR68 (1:600, Zen-Bio, Cat No.370126), p-Akt (1:1200, Abcam, Cat No. ab192623), Akt (1:1200, Abcam, Cat No. ab179463), NRF2 (1:1000, Proteintech Group, Cat No.CL594-16396), β-actin (1:8000, Proteintech Group, Cat No. 20536-1-AP), and histone H3 (1:3000, Proteintech Group, Cat No. 17168-1-AP). Secondary antibodies were conjugated to horseradish peroxidase and detected using an ECL system. Protein bands were visualized and analyzed using ImageJ Software (Version 1.53c). Target protein levels were normalized to their respective loading controls (β-actin or Histone H3)

### Pharmacological Interventions

#### In Vitro Drug Treatments

PC12 cells were exposed to pharmacological agents during oxygen-glucose deprivation/reperfusion (OGD/R) as follows:

- GPR68 activation: 10 µM MS48107 (MedChemExpress, Cat No.HY-134494) in culture medium was administered throughout the OGD/R process, based on preliminary dose-response experiments showing maximal Akt phosphorylation at this concentration.
- PI3K inhibition: 1 µM LY294002 (MedChemExpress, Cat No.HY-10108) was co-applied with MS48107 to block downstream signaling.

#### In Vivo Drug Administrations

Intrathecal OGM (MedChemExpress, Cat No.HY-154954) delivery:

Male SD rats (250–300 g) were anesthetized with 2% isoflurane and positioned in ventral recumbency. Using aseptic technique, a 30-gauge spinal needle was inserted into the L5-L6 intervertebral space until tail flick reflex confirmed proper intrathecal placement. OGM dissolved in artificial cerebrospinalfluid (CSF: NaCl 147 mM, KCl 2.7 mM, CaCl₂ 1.2 mM) was infused at 10 µL/min via microsyringe pump, delivering 4 nmol/kg (10 µM solution adjusted for rat CSF volume ~ 25 µL/100 g body weight). Injections commenced 12 h pre-ischemia and repeated every 24 h until sacrifice at 72 h post-reperfusion.

#### Oral Lorazepam Administration

Lorazepam (Atlantic LabCorp.Ltd) was suspended in sterile corn oil at 1 mg/mL and administered by oral gavage using a 18-gauge stainless steel feeding needle. The dosing regimen consisted of 5 mg/kg [34] given 12 h before ischemia induction, followed by twice-daily maintenance doses (every 12 h) until tissue collection, ensuring plasma concentrations above 200 ng/mL based on published pharmacokinetics (T₁/β = 10.5 h in rats).Control groups received equivalent volumes of vehicle (0.1% DMSO for intrathecal, corn oil for oral).

### Hematoxylin and Eosin (H&E) Staining

Paraffin-embedded tissue sections (4 μm thick) were deparaffinized in xylene, rehydrated through graded ethanol series, and stained with hematoxylin (5 min). After differentiation in 1% acid alcohol and bluing in 0.2% ammonia water, sections were counterstained with eosin Y (1 min). Dehydration was performed through ascending ethanol gradients, followed by xylene clearance. Sections were mounted with neutral balsam and observed under a light microscope (OLYMPUS).

### Nissl Staining and Quantitative Analysis

Tissue sections were stained with 0.1% Cresyl Violet solution for 5 min at RT, followed by brief differentiation in 95% ethanol containing 0.05% glacial acetic acid. After dehydration and clearing, slides were mounted for imaging. Bright-field images of lumbar spinal cord ventral horn were captured at 10× and 20× magnification. For Nissl body quantification, ImageJ Software (Version 1.53c) was used to manually outline cellular regions of interest (ROI) containing intact cells. The Cresyl Violet signal was isolated by color thresholding (purple granular staining), and the percentage of Nissl-positive area within each ROI was calculated relative to the total cytoplasmic area. Optical density of Nissl staining was measured after subtracting background signals from adjacent white matter. Data from five animals per group (three sections per animal) were statistically analyzed by one-way ANOVA and expressed as mean ± SEM.

### Immunohistochemistry (IHC)

Lumbar spinal cord tissues were fixed in 4% paraformaldehyde for 24 h, processed for paraffin embedding, and sectioned at 4–5 μm. After deparaffinization and antigen retrieval, endogenous peroxidase activity was blocked with 3% H₂O₂. Sections were incubated with 5% BSA for 1 h, followed by primary antibody against GPR68 (1:600, Zen-Bio, Cat No.370126) overnight at 4 °C and HRP-conjugated secondary antibody for 1 h at room temperature. DAB substrate was used for visualization, with hematoxylin counterstaining. GPR68 expression was evaluated using the H-SCORE method:

Staining intensity scoring (per cell):

- 0: No staining.
- 1: Weak cytoplasmic staining (light brown).
- 2: Moderate staining (distinct brown granules).
- 3: Strong staining (dark brown, granular pattern).

Percentage estimation of positive cells at each intensity within the ventral horn ROI.

$$\begin{array}{l}\mathrm H-\mathrm{Score}\;=\;(1\times\mathrm{Percentage}\;\mathrm{of}\;\mathrm{Weak}\;\mathrm{cytoplasmic}\;\mathrm{staining})\\\;\;\;\;\;\;\;\;\;\;\;\;\;\;\;\;\;\;+\;(2\times\mathrm{Percentage}\;\mathrm{of}\;\mathrm{Moderate}\;\mathrm{staining})\\\;\;\;\;\;\;\;\;\;\;\;\;\;\;\;\;\;\;+\;(3\times\mathrm{Percentage}\;\mathrm{of}\;\mathrm{Strong}\;\mathrm{staining}).\end{array}$$

Yielding a theoretical range of 0–300%.

Three non-overlapping fields per section (3 sections/animal, 5 animals/group) were analyzed independently by two blinded observers. Inter-observer correlation coefficient (ICC) > 0.85 was required for data inclusion. Results were expressed as mean ± SD and analyzed by Kruskal-Wallis test.

### Immunofluorescence Staining and Quantification

PBS-washed cells on coverslips were fixed with 4% paraformaldehyde (15 min, RT) and permeabilized with 0.1% Triton X-100 (5 min, RT). After blocking with 5% BSA (1 h, RT), cells were incubated overnight at 4 °C with primary antibodies: mouse anti-β-tubulin (1:1000, Proteintech Group, Cat No.80713-1-RR) and rabbit anti-NRF2 (1:1000, Proteintech Group, Cat No.CL594-16396). Subsequently, cells were incubated with secondary antibodies: PE-conjugated goat anti-mouse IgG (H + L) (for β-tubulin, 1:500) and FITC-conjugated goat anti-rabbit IgG (H + L) (for NRF2, 1:500) for 1 h at RT in the dark. Nuclei were counterstained with DAPI (5 µg/mL, 10 min).

Images were acquired using a confocal laser scanning microscope (OLYMPUS) identical exposure settings.

### Nuclear-to-Cytoplasmic (N/C) Ratio Quantification

The N/C ratio of NRF2 was calculated by dividing the mean fluorescence intensity (MFI) of NRF2 in nuclei (DAPI-defined regions) by that in cytoplasm (β-tubulin-outlined areas excluding nuclei), using ImageJ Software (Version 1.53c). Background signals from cell-free regions were subtracted. At least 30 cells per group were analyzed across three independent experiments. Data are expressed as mean ± SD, and statistical significance was determined by one-way ANOVA with Tukey’s post-hoc test (GraphPad Prism v3.2).

### Detection of MDA and GSSG/T-GSH

#### MDA Detection

MDA levels were measured using a MDA Assay Kit (Beyotime) according to the manufacturer’s instructions. Briefly, samples were prepared by adding Assay Buffer and processed through heating and centrifugation. The reaction was initiated by adding specific reagents and heating, followed by measuring the absorbance at 532 nm.

#### GSSG/T-GSH Detection

GSSG and T-GSH levels were determined using a Total Glutathione/Oxidized Glutathione Assay Kit (Servicebio) according to the manufacturer’s instructions. Samples were processed with protein removal reagent and centrifugation. For T-GSH detection, samples were reacted with a working solution containing glutathione reductase and DTNB, and absorbance was measured at 412 nm. For GSSG detection, GSH was removed using a scavenger, and the remaining GSSG was converted to GSH for detection.

### Statistical Analysis

Data were expressed as mean ± standard deviation (SD). Statistical analyses were performed using GraphPad Prism software (Version 10.3.2). Comparisons between two groups were analyzed using unpaired Student’s t-tests. For multiple comparisons, one-way ANOVA followed by Tukey’s post hoc test was used. A p-value < 0.05 was considered statistically significant. In all experiments, *n* = 3.

## Results

### OGD/R Triggers Ferroptosis with Concomitant GPR68 Downregulation In Vitro

**Fig. 1:**
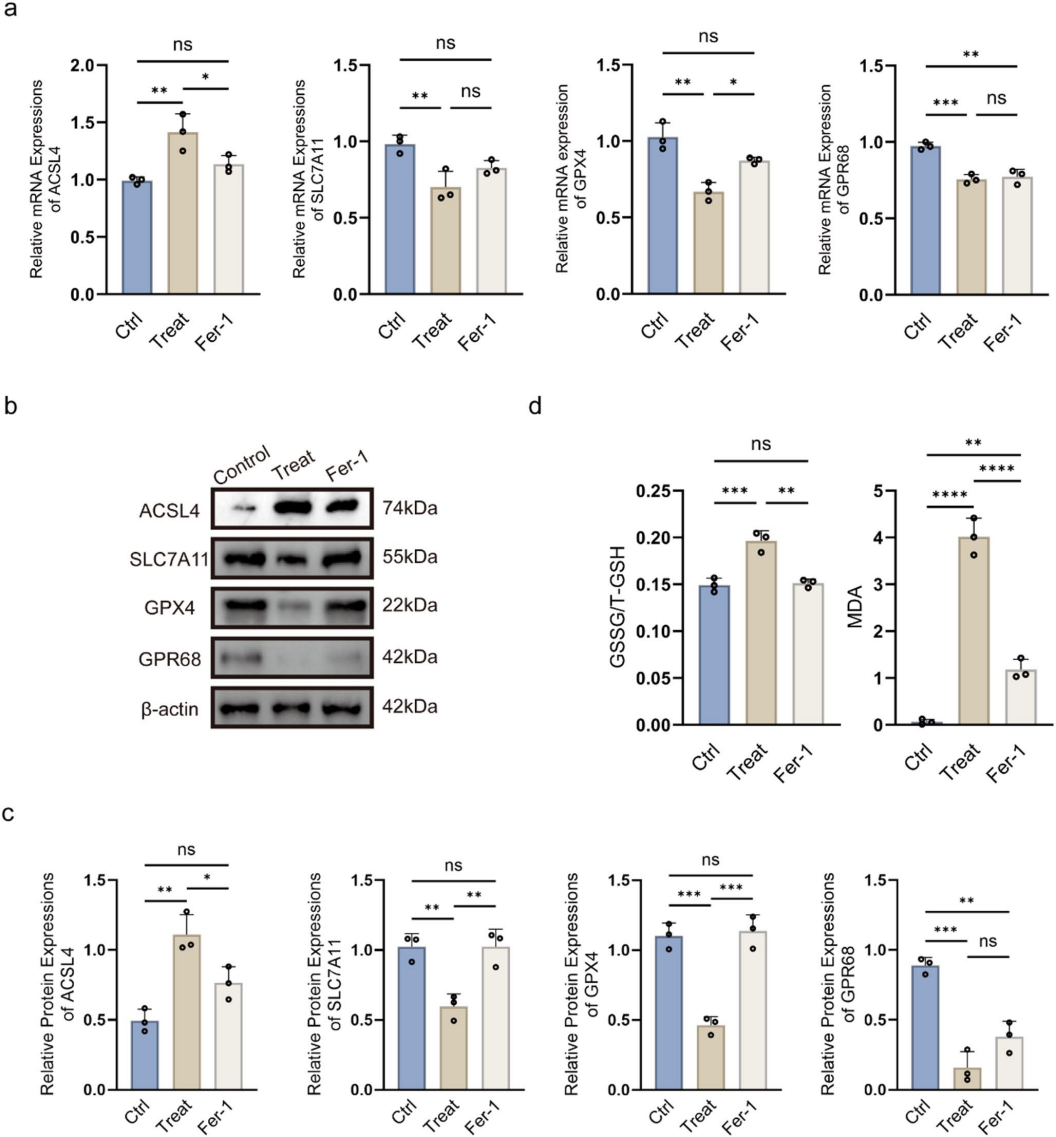
OGD/R triggers ferroptosis and suppresses GPR68 in PC12 cells. **a** qPCR analysis of ferroptosis-related genes (ACSL4, SLC7A11, GPX4). **b** Representative western blots. **c** Quantitative protein expression normalized to β-actin. **d** Oxidative stress markers (GSSG/T-GSH ratio and MDA levels). Data are mean ± SD; *p < 0.05, **p < 0.01, ***p < 0.001, ****p < 0.0001 (*n* = 3, one-way ANOVA)

qPCR analysis demonstrated that OGD/R significantly altered mRNA levels of ferroptosis-related markers in rat PC12 cells (Fig. 1a). Western blot (Fig. 1b) further confirmed these changes at the protein level: ACSL4 was upregulated (*p* < 0.01), while SLC7A11 (*p* < 0.01) and GPX4 (*p* < 0.001) were downregulated compared to the control group (Fig. 1c). OGD/R also increased oxidative stress, as evidenced by elevated GSSG/T-GSH ratio (*p* < 0.001) and MDA levels (*p* < 0.0001) (Fig. 1d). Co-treatment with Ferrostatin-1 partially reversed the OGD/R-induced changes in ACSL4 (*p* < 0.05), SLC7A11 (*p* < 0.01) and GPX4 (*p* < 0.001). Additionally, GPR68 protein expression was markedly reduced following OGD/R (*p* < 0.001; Fig. 1c).

### GPR68 Activation Attenuates Ferroptosis Through Akt/Nrf2 Signaling in OGD/R-treated Cells

Bioinformatic KEGG analysis of spinal cord injury transcriptomes (rat/mouse) highlighted PI3K/Akt pathway enrichment in GPR68-associated differential genes (Supplementary Fig. S1). To validate this, we examined Akt/Nrf2 signaling and ferroptosis markers under OGD/R with pharmacological or genetic interventions.

**Fig. 2:**
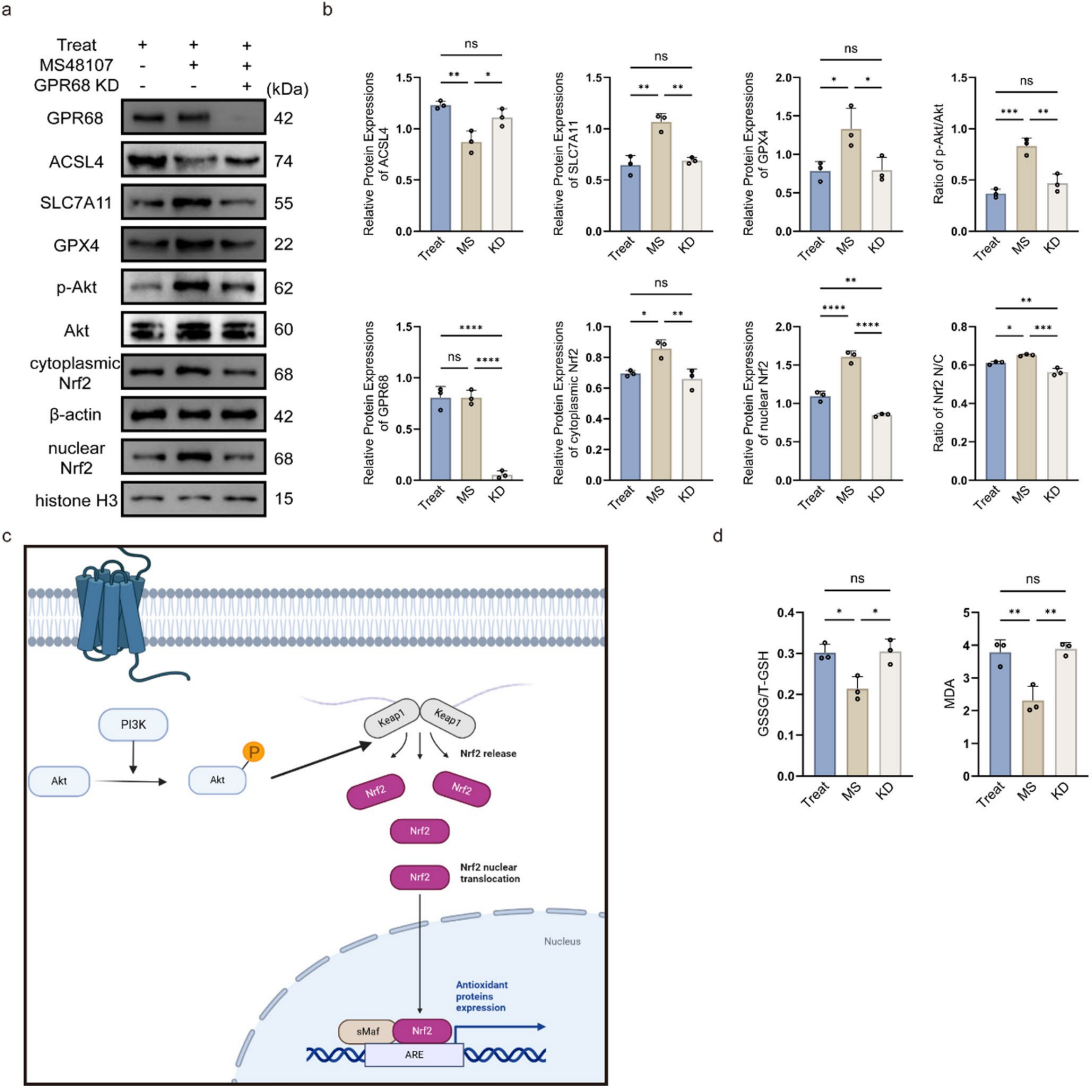
GPR68-mediated ferroptosis regulation through Akt/Nrf2 pathway. **a** Representative western blots. **b** Quantitative protein expression normalized to β-actin or histone H3(nuclear Nrf2). **c** Schematic mechanism. **d** Oxidative stress markers (GSSG/T-GSH ratio and MDA levels). Data are mean ± SD; *p < 0.05, **p < 0.01, ***p < 0.001, ****p < 0.0001 (*n* = 3, one-way ANOVA)

Western blot revealed that GPR68 activation by MS48107 in OGD/R-treated cells significantly enhanced Akt phosphorylation (p-Akt, Ser473; *p* < 0.001 vs. OGD/R) and increased the nuclear/cytoplasmic Nrf2 ratio (*p* < 0.05; normalized to histone H3 and β-actin, Fig. 2a-b). This was accompanied by downregulation of the pro-ferroptosis protein ACSL4 (*p* < 0.01) and upregulation of anti-ferroptosis proteins SLC7A11 (*p* < 0.01) and GPX4 (*p* < 0.05). Conversely, GPR68 knockdown (siRNA) reversed these effects (Fig. 2a-b). Oxidative stress markers (GSSG/T-GSH and MDA) were similarly rescued by MS48107 but abolished by siRNA (Fig. 2d). A mechanistic diagram (Fig. 2c) integrates these findings, illustrating that GPR68 activation promotes p-Akt-dependent Nrf2 nuclear translocation, leading to suppression of ferroptosis via SLC7A11/GPX4 upregulation and lipid peroxidation mitigation.

### Akt Inhibition Abolishes GPR68-mediated Ferroptosis Protection in OGD/R Injury

**Fig. 3:**
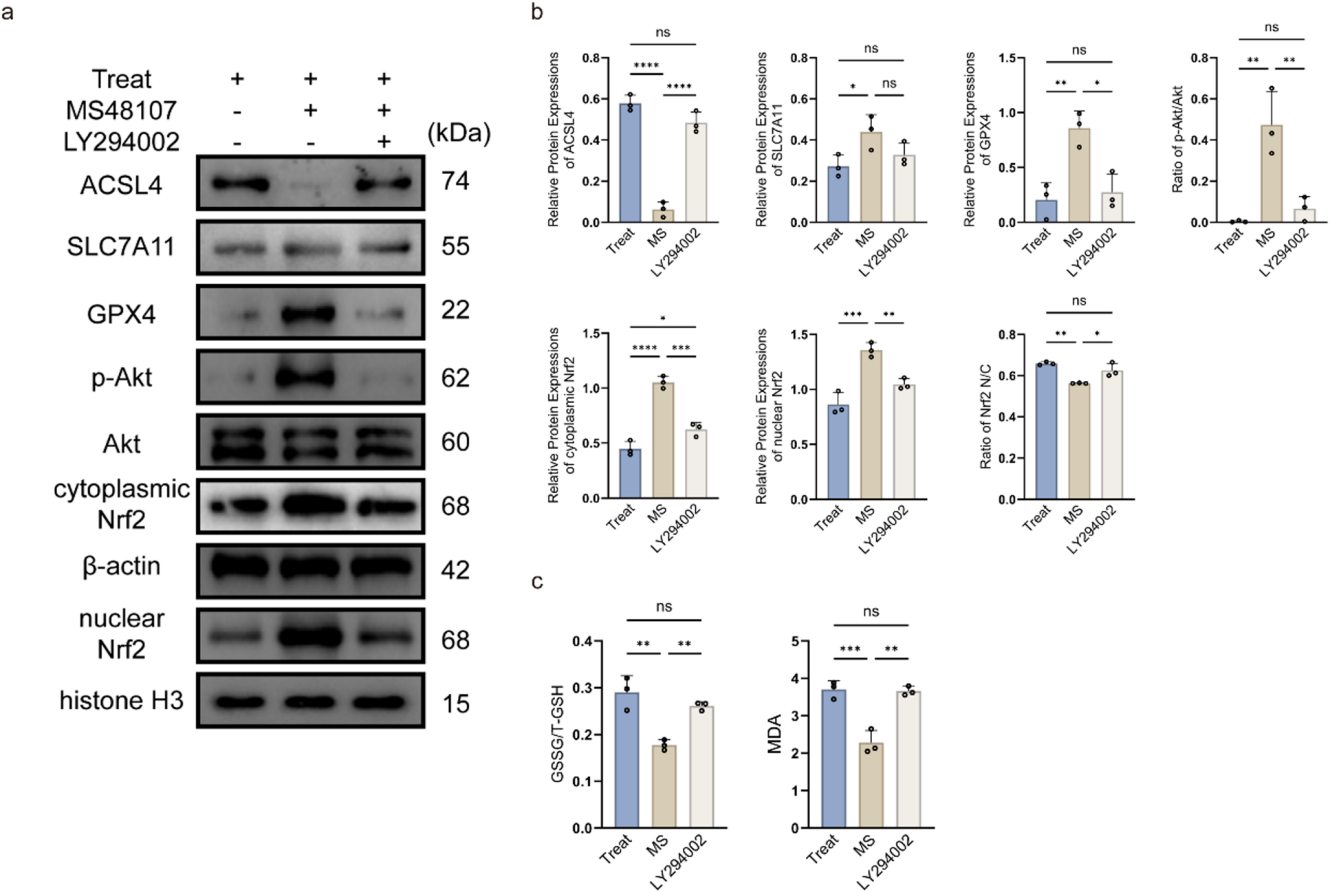
Akt dependency of GPR68 anti-ferroptosis effects. **a** Representative western blots. **b** Quantitative protein expression normalized to β-actin or histone H3(nuclear Nrf2). **c** Oxidative stress markers (GSSG/T-GSH ratio and MDA levels). Data are mean ± SD; *p < 0.05, **p < 0.01, ***p < 0.001, ****p < 0.0001 (*n* = 3, one-way ANOVA)

To validate the necessity of Akt in GPR68 signaling, we combined the GPR68 agonist MS48107 with the Akt inhibitor LY294002 in OGD/R-treated cells. While MS48107 alone enhanced Akt phosphorylation (p-Akt, Ser473; *p* < 0.01 vs. OGD/R) and nuclear Nrf2 translocation (*p* < 0.01), these effects were completely abrogated by LY294002 (Fig. 3a-b). Consequently, the anti-ferroptosis effects of MS48107—suppression of ACSL4 (*p* < 0.0001) and upregulation of SLC7A11 (*p* < 0.05) and GPX4 (*p* < 0.01)—were reversed to OGD/R levels (Fig. 3a-b). LY294002 also negated the antioxidant capacity of MS48107, elevating GSSG/T-GSH ratio (*p* < 0.01) and MDA levels (*p* < 0.01; Fig. 3c).

### GPR68 Activation Triggers Akt-dependent Nrf2 Nuclear Translocation Under Basal Conditions

**Fig. 4:**
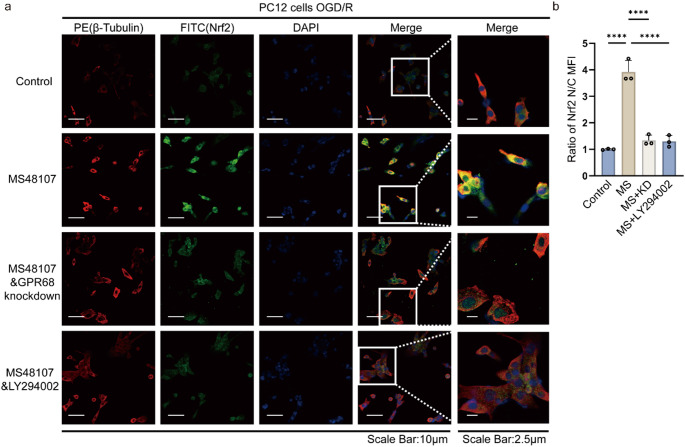
GPR68 controls basal Nrf2 nuclear translocation via Akt. **a** Immunofluorescence images of Nrf2 (green) in PC12 cells. **b** Quantification of nuclear/cytoplasmic Nrf2 ratio. Data are mean ± SD; ****p < 0.0001 (*n* = 5, one-way ANOVA)

Immunofluorescence staining demonstrated that GPR68 activation by MS48107 in untreated cells robustly promoted Nrf2 nuclear accumulation (green fluorescence colocalized with DAPI; *p* < 0.0001 vs. control; Fig. 4a-b). This effect was fully abolished by GPR68 knockdown (siRNA; *p* < 0.0001) and Akt inhibition (LY294002; *p* < 0.0001), confirming the distinct roles of GPR68 and Akt in mediating Nrf2 translocation (Fig. 4a-b). β-Tubulin (red) and DAPI (blue) confirmed intact cellular architecture.

### Spinal Cord Ischemia-Reperfusion Injury Triggers Time-Dependent Ferroptosis and GPR68 Downregulation in Rats

**Fig. 5:**
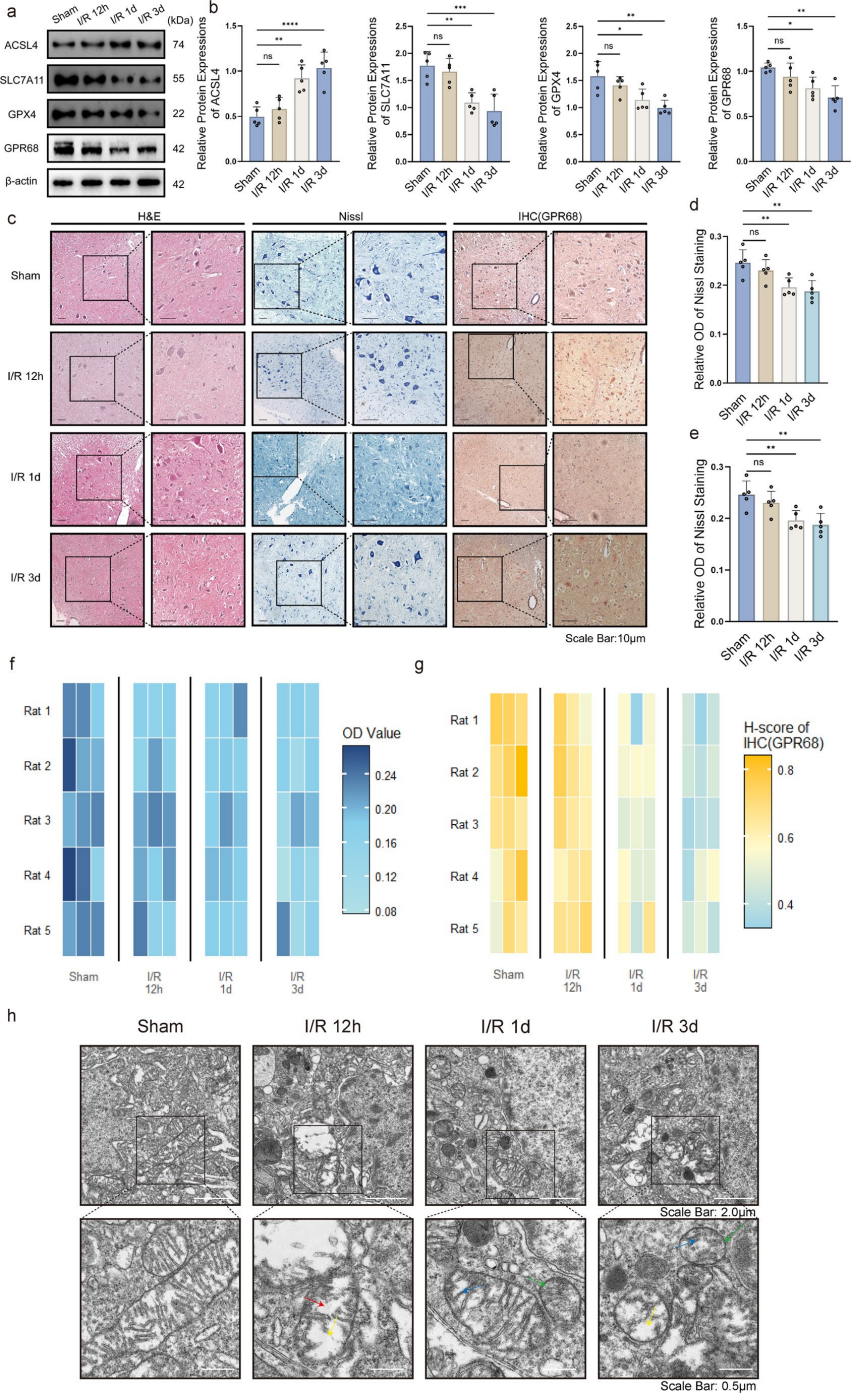
Temporal dynamics of ferroptosis and GPR68 in spinal cord I/R injury. **a** Western blots of ACSL4, SLC7A11, GPX4, and GPR68 at 12 h/1d/3d post-I/R. **b** Protein quantification. **c** HE staining (pathological changes), Nissl staining (neuronal integrity) and GPR68 IHC (brown signal). **d-e** Quantitative of Nissl body loss and GPR68 reduction. **f** Quantitative heatmap of Nissl staining. **g** Quantitative heatmap of IHC (GPR68). Data are mean ± SD; *p < 0.05, **p < 0.01, **p < 0.001 vs. Sham (*n* = 5, one-way ANOVA). **h** Ultrastructural changes in neuronal mitochondria post spinal cord I/R (TEM)

Using a rat spinal cord ischemia-reperfusion (I/R) model, we observed time-dependent ferroptosis activation and GPR68 downregulation. Western blot revealed progressive upregulation of ACSL4 (3d: *p* < 0.0001 vs. Sham) alongside downregulation of SLC7A11 (3d: *p* < 0.001) and GPX4 (3d: *p* < 0.01) (Fig. 5a-b). GPR68 expression was markedly reduced post-I/R (3d: *p* < 0.01; Fig. 5a-b).

Histologically, HE staining exhibited escalating pathological alterations:

- 12 h: Early neuronal shrinkage, mild cytoplasmic eosinophilia, scattered vacuolation in gray matter.
- 1d: Pronounced neuronal loss, nuclear pyknosis, disrupted parenchymal architecture with enlarged perineuronal spaces.
- 3d: Severe necrosis, inflammatory infiltration (lymphocytes), and hemorrhagic foci in both gray and white matter (Fig. 5c).

Nissl staining corroborated neuronal damage, showing progressive Nissl body dissolution (3d vs. Sham: *p* < 0.01; Fig. 5d). IHC confirmed time-dependent GPR68 reduction in motor neurons (3d vs. Sham: *p* < 0.001; Fig. 5e), visualized as diminished cytoplasmic/membrane staining (Fig. 5c). Quantitative heatmaps highlighted the inverse correlation between Nissl loss and GPR68 depletion (Fig. 5f-g). The peak ferroptosis and GPR68 suppression at 3 d post-I/R justified selecting this timepoint for therapeutic interventions.

Transmission electron microscopy (TEM) revealed characteristic ferroptotic mitochondrial damage in spinal cord tissues post-I/R: Sham group mitochondria showed intact structure with clearly visible cristae, while I/R groups exhibited progressive alterations—early mitochondrial swelling (red arrows) at 12 h, followed by late-stage ferroptotic features at 1d-3d, including shrunken rounded/oval mitochondria with abnormally increased membrane density (green arrows), vacuolization indicating membrane integrity loss (yellow arrows), and cristae dissolution/disappearance (blue arrows; distinct from apoptosis), collectively corroborating ferroptosis progression alongside aforementioned experimental findings. High-resolution images appear in Supplementary File 2.

### Pharmacological activation of GPR68 by Lorazepam attenuates ferroptosis through Akt/Nrf2 signaling in spinal cord I/R injury

**Fig. 6:**
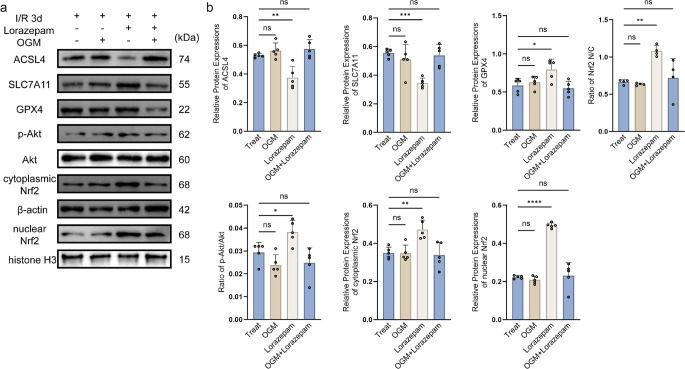
Lorazepam rescues spinal cord I/R injury via GPR68/Akt/Nrf2 axis. **a** Representative western blots. **b** Quantitative protein expression normalized to β-actin or histone H3(nuclear Nrf2). Data are mean ± SD; *p < 0.05, **p < 0.01, ***p < 0.001, ****p < 0.0001 (*n* = 5, one-way ANOVA)

In the rat spinal cord I/R 3 d model, pharmacological targeting of GPR68 demonstrated its critical role in regulating ferroptosis (Fig. 6a). Treatment with Lorazepam significantly reduced ACSL4 (*p* < 0.01), upregulated SLC7A11 (*p* < 0.001) and GPX4 (*p* < 0.05), and enhanced Akt phosphorylation (*p* < 0.05) alongside nuclear Nrf2 accumulation (*p* < 0.01). In contrast, OGM, a selective GPR68 inhibitor, alone did not exhibit statistically significant effects on ferroptosis markers or Akt/Nrf2 signaling (all *p* > 0.05) compared to the I/R group. Strikingly, co-administration of OGM and Lorazepam completely abolished the protective effects of Lorazepam (Fig. 6b).

## Discussion

This study elucidates a novel mechanistic link between acidosis-sensing GPR68 and ferroptosis regulation in spinal cord ischemia-reperfusion injury (SCIRI). Our findings demonstrate that GPR68 downregulation exacerbates ferroptosis damage by disrupting the PI3K/Akt/Nrf2 antioxidant axis, while pharmacological activation of GPR68—via the benzodiazepine Lorazepam—rescues neuronal viability through this pathway. These data advance our understanding of pH-sensitive GPCRs in ferroptosis pathophysiology and offer a translational strategy for repurposing clinically approved drugs to mitigate SCIRI.

### GPR68 as a pH-Sensitive Ferroptosis Regulator

The role of GPR68 in sensing microenvironmental acidosis has been documented in inflammatory and ischemic contexts, yet its involvement in ferroptosis remained unexplored. Our in vitro and in vivo models reveal that GPR68 expression inversely correlates with ferroptosis markers (ACSL4, SLC7A11, GPX4) during OGD/R and spinal I/R. This aligns with the known role of acidosis in aggravating lipid peroxidation, but uniquely identifies GPR68 as a molecular bridge linking extracellular pH changes to intracellular ferroptosis machinery. The complete reversal of MS48107-mediated protection by GPR68 siRNA or OGM underscores the specificity of this receptor in ferroptosis suppression.

### Hierarchical PI3K/Akt/Nrf2 Signaling Underlies GPR68’s Anti-Ferroptosis Effects

We provide compelling evidence that GPR68 activates PI3K/Akt signaling to drive Nrf2 nuclear translocation, a critical antioxidant mechanism. The requirement for Akt in this cascade is unequivocally demonstrated by LY294002 abolishing both Nrf2 activation and ferroptosis mitigation. Notably, GPR68 activation under basal conditions robustly induced Nrf2 nuclear accumulation, suggesting tonic receptor activity even in non-ischemic environments. This expands the paradigm of GPCR-mediated cytoprotection, which traditionally focuses on canonical Gαq/11 or Gαs pathways, by highlighting a distinct GPR68-PI3K/Akt-Nrf2 axis.

### Translational Implications of Lorazepam as a GPR68-Targeted Therapy

The identification of Lorazepam—a widely used anxiolytic—as a functional GPR68 agonist represents a key translational advance. Our data show that Lorazepam rescues GPR68 expression, reactivates Akt/Nrf2 signaling, and mitigates ferroptosis in vivo. Importantly, the complete negation of its effects by OGM confirms GPR68-dependent action, addressing concerns about benzodiazepine neuroprotection via GABA receptors. This finding aligns with emerging interest in drug repurposing for ischemic injury, offering a clinically deployable strategy to limit SCIRI complications.

### Limitations and Future Directions

While our study establishes GPR68 as a central ferroptosis regulator, several questions remain. First, the precise molecular mechanism of GPR68 downregulation during I/R—whether transcriptional repression, protein degradation, or pH-dependent internalization—requires further investigation. Second, the contribution of non-neuronal cells (e.g., astrocytes, microglia) expressing GPR68 to ferroptosis regulation remains unaddressed. Third, although Lorazepam showed efficacy in our model, its long-term effects on spinal cord recovery and potential off-target actions merit evaluation in chronic injury models.

## Conclusion

Collectively, we unveil GPR68 as a critical sensor of ischemic acidosis that orchestrates ferroptosis resistance via PI3K/Akt/Nrf2 signaling. The therapeutic efficacy of Lorazepam in preserving spinal cord integrity underscores the clinical potential of targeting pH-sensing GPCRs in ischemia-reperfusion pathologies. Future studies should explore combinatorial therapies augmenting GPR68 signaling alongside existing neuroprotective regimens to optimize outcomes in spinal surgeries or traumatic injuries.

## Supplementary Information

Below is the link to the electronic supplementary material.

Supplementary Material 1 (DOCX 618 KB)

Supplementary Material 2 (ZIP 21.9 MB)

## Data Availability

No datasets were generated or analysed during the current study.
