# Supplementary material for: Pharmacological Activation of GPR68 Attenuates Ferroptosis in Spinal Cord Ischemia/Reperfusion Injury Through PI3K/Akt-Mediated Nrf2 Antioxidant Pathway
Source: Inflammation. 2025 Jun 18;48(6):4289–301. doi: 10.1007/s10753-025-02326-0 (PMC12722321; doi:10.1007/s10753-025-02326-0)
Supplement: Supplementary file 1 — Supplementary Material 1 (DOCX 618 KB) [file 10753_2025_2326_MOESM1_ESM.docx]

First Author: **Ruitong Yang**

Second Affiliated Hospital of Xi’an Jiaotong University,

No.157 Xiwu Road, Xi’an, 710000, Shaanxi, China

Email: 2384883911@qq.com

Corresponding Author: **Fengtao Li**

Second Affiliated Hospital of Xi’an Jiaotong University,

No.157 Xiwu Road, Xi’an, 710000, Shaanxi, China

Email: [lft369@163.com](mailto:lft369@163.com)

Subject: Submission of manuscript entitled "**Pharmacological Activation of GPR68 Attenuates Ferroptosis in Spinal Cord Ischemia/Reperfusion Injury through PI3K/Akt-Mediated Nrf2 Antioxidant Pathway**"


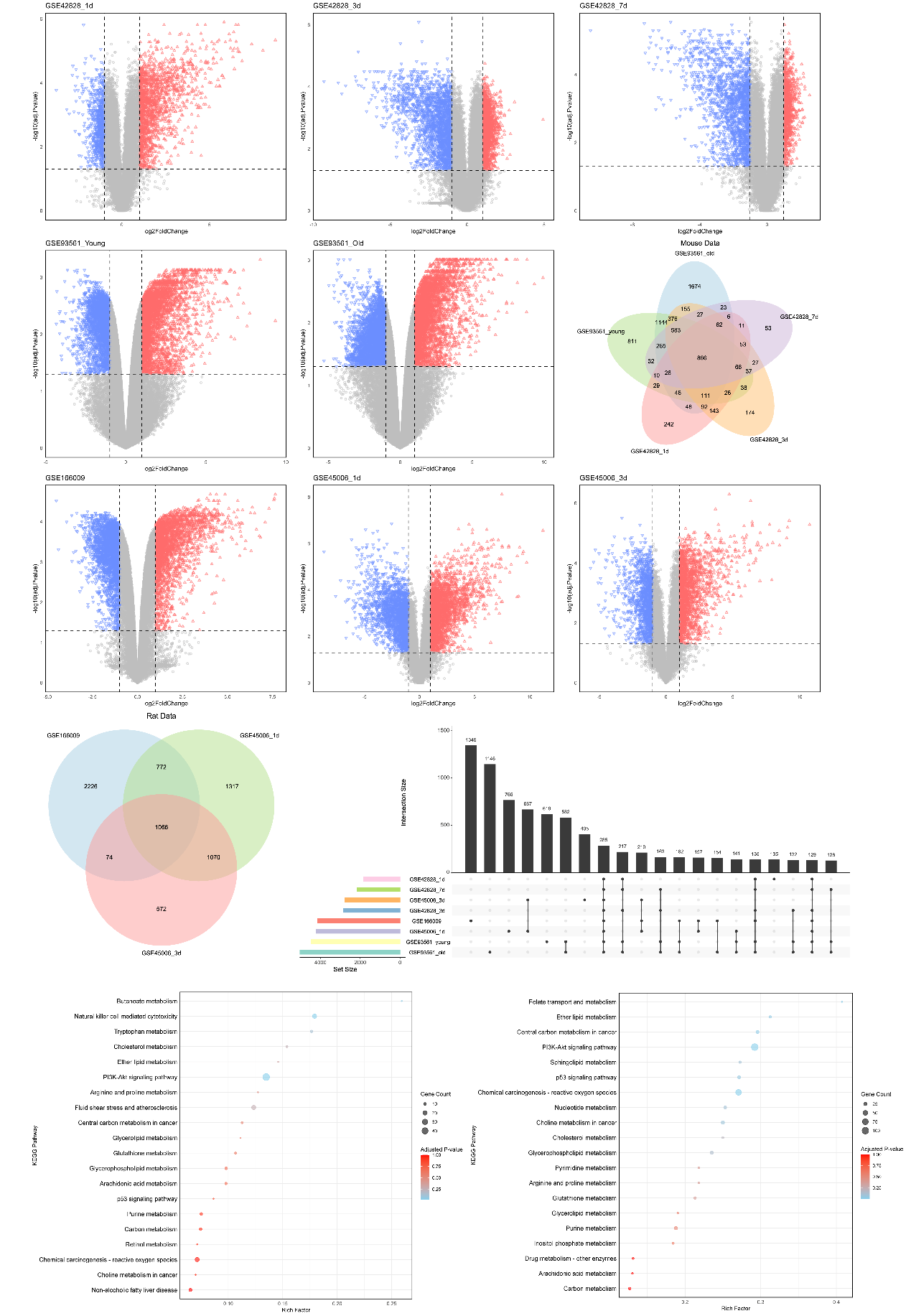
Fig.S 1
